# Effect of Modified Formula Radix Hedysari on the Amplification Effect during Peripheral Nerve Regeneration

**DOI:** 10.1155/2013/647982

**Published:** 2013-02-24

**Authors:** Zhi Yong Wang, Pei Xun Zhang, Na Han, Yu Hui Kou, Xiao Feng Yin, Bao Guo Jiang

**Affiliations:** Department of Trauma and Orthopeadics, Peking University People's Hospital, Beijing 100044, China

## Abstract

Many studies have demonstrated a compensatory amplification phenomenon during nerve regeneration. When a relatively fine nerve is used as a donor to connect to a distal nerve after transection, the donor nerve regenerates more collaterals than its own fibers, which extend to the distal stump, grow into distal endoneurial tubes, and finally reach and dominate the target organs. This is known as the amplification phenomenon. In this study, we investigated the amplification phenomenon in rats treated with Modified Formula Radix Hedysari (MFRH) as adjuvant therapy for 12 weeks. The rats were divided into three groups at random (six animals in each group). In the model group and the treatment group, the proximal common peroneal nerve was used as a donor nerve to connect to the distal tibial nerve. Rats in the normal group did not undergo surgery. After surgery, the treatment group was administered MFRH as systemic therapy, while the model group and the normal group were not given treatment. The results demonstrated that the nerve conduction velocity, the fiber diameter, the axon diameter, the number of regenerating nerve fibers, and the amplification ratio were better in the treatment group than in the model group, suggesting that MFRH promoted the nerve amplification effect.

## 1. Introduction

Previous studies have shown that single axons can grow several new lateral buds in the initial stages of nerve regeneration. The total number of lateral buds that the proximal fibers grew was significantly more than the number of distal endoneurial tubes [1, 2]. Using fewer proximal fibers to bridge the distal nerve enabled the amplification phenomenon to be achieved during nerve regeneration, and the maximum amplification ratio for nerve regeneration was about 3.3. However, the number of nerve fibers generated by amplification is not sufficient for neurological function recovery. Previous studies on nerve amplification phenomena have focused only on natural nerve growth and not on the effect of adjuvant therapy. To address this issue, we investigated whether Traditional Chinese Medicine as an adjuvant treatment after surgical operation can promote the amplification effect by inducing increased lateral bud growth on axons. We used the Traditional Chinese Medicine, Modified Formula Radix Hedysari (MFRH), which consists of Radix Hedysari, *Epimedium* and *Lumbricus*. Previous studies have shown that Hedysari Polysaccharides and *Lumbricus* extract could effectively promote peripheral nerve regeneration after nerve clamping injury and could significantly improve the recovery of nerve function [3, 4]. In this study, we further investigated the effect of MFRH on the amplification phenomenon.

## 2. Materials and Methods

### 2.1. Drug Preparation

In the MFRH prescription, Hedysari and *Epimedium* were produced in and purchased from Gansu Province, China. *Lumbricus*, produced in Guangdong Province, China, was purchased from Beijing, China. Hedysari, *Epimedium*, and *Lumbricus* were decocted according to a traditional decocting method, as follows. They were first decocted with distilled water for 2 h and then for 1 h, following that the mixed solution was concentrated into 1 g/mL (equivalent to dry weight of crude drug) and stored at 4°C until use.

### 2.2. Animals

Male Sprague-Dawley rats weighing 200–250 g were maintained under specific pathogen-free laboratory conditions on a 12 h light/dark cycle with free access to pellet food and water. The rats were separated into three groups at random (six animals in each group). Every effort was made to minimize animal suffering and reduce the number of animals used, according to the Chinese guidelines for the care and use of laboratory animals.

### 2.3. Materials

The materials used were chitin biological absorbable tubes (ether-free chitin biological tubes; length, 8 mm; wall thickness, 0.2 mm; inner diameter, 1.5 mm), a synergy electrophysiological instrument, a Leica dissecting microscope, a Leica tissue embedding machine, and a Leica image collection and analysis system.

### 2.4. Surgical Procedures

**Figure 1:**
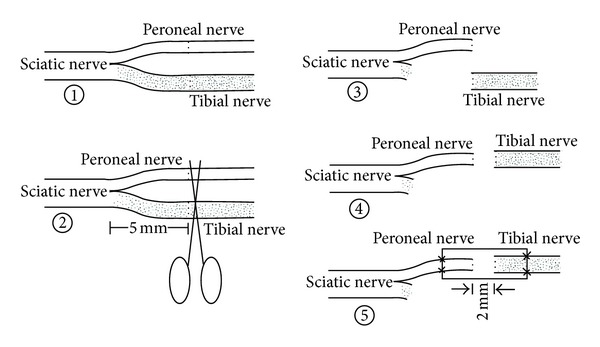
Surgical procedures.

Surgical procedures were performed in a specific pathogen-free animal laboratory using a microsurgical technique. Rats in the model group and the treatment group were anesthetized with sodium pentobarbital (30 mg/kg i.p.). Following anesthesia, the right limbs were treated in a sterile manner. The sciatic nerve and its two main branches (the common peroneal nerve and the tibial nerve) were exposed. The common peroneal nerve and the tibial nerve were transected at 5 mm distal to the bifurcation. The proximal stump of the tibial nerve and the distal stump of the common peroneal nerve were ligated with 10-0 nylon sutures and stitched to the adjacent muscle. The proximal stump of the common peroneal nerve served as the donor nerve and was fixed to the distal stump of the tibial nerve. For this, we used chitin biological absorbable conduits to create an artificial nerve graft; the conduits consist of a polysaccharide shell that showed satisfactory biocompatibility and degradation characteristics. 10-0 nylon microsutures were used. The gap between the two nerve segments was kept at 2 mm. Subsequently, the muscle incision was sutured and the wound closed using 4-0 nylon sutures (Figure 1). The untransected tibial nerves served as the normal group. Rats were then placed back into the cages in which they were raised, and the nerves were removed for examination 12 weeks after operation.

### 2.5. Treatment Method

Starting from the first day after surgery, each rat in the normal group and the model group was treated with 2 mL 0.9% NaCl by oral gavage once daily, and each rat in the treatment group was treated with 2 mL MFRH liquid (1 g/mL) in the same manner at the same time every day. The duration of oral gavage was 12 weeks.

## 3. Analytical Procedures

### 3.1. General Observations

After surgical operation, the general health of the animals was regularly observed, including the degree of wound healing, the activities of the operated limbs, ulcer formation, and rotten situation on feet caused by self-biting toes.

### 3.2. Walking Track Analysis

Walking track analysis was performed for the animals in each group at 12 weeks after surgery. Animals were allowed conditioning trials in a confined walking track (10 × 60 cm) darkened at one end. White paper with the appropriate dimensions was placed on the bottom of the track. The rat's hind limbs were dipped into black ink before the animal was placed at the entrance of the walking track. Foot prints appeared immediately on the paper after the rat walked down the track. Prints for measurement were chosen at the time of walking, based on clarity and completeness at a point when the rat was walking briskly. Animals in the normal group underwent the same procedure. 

Paired footprint parameters for print length (distance from heel to toe, PL), toe spread (distance from first to fifth toe, TS), and intermediary toe spread (distance from second to fourth toe, IT) were recorded for the left normal control foot (NPL, NTS, and NIT) and the corresponding right experimental foot (EPL, ETS, and EIT) for each rat.

Tibial function index (TFI) was calculated according to the Bain-Mackinnon-Hunter formula:

(1) TFI=−37.2([EPL−NPL]NPL)+104.4([ETS−NTS]NTS)+45.6([EIT−NIT]NIT)−8.8.

### 3.3. Electrophysiological Examination

Electrophysiological examination was conducted 12 weeks after surgery prior to sacrifice of the animals. The repaired tibial nerve was exposed, and stimulating bipolar electrodes were placed proximal and distal to the repair site in each group. The recording electrode was placed in the gastrocnemius muscle, while the ground electrode was placed in subcutaneous tissue between the stimulating and recording electrodes. Rectangular pulses (duration 0.1 ms, 0.9 mA, 10 Hz, 6 continual stimuli) were used. Upon the stimulation of the repaired tibial nerves, the nerve conduction velocity (NCV; m/s) was obtained semiautomatically by dividing the distance between the two stimulating sites by the difference in the conduction time. 

### 3.4. Histological Analysis

**Figure 2:**
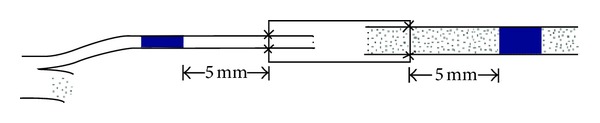
The excised specimen.

After electrophysiological examination, the entire nerve, including the repaired segment, was removed from each rat. Tissues were then harvested and fixed in 4% paraformaldehyde in 0.1 M phosphate buffer for 24 h at 4°C. The nerves were then rinsed twice in water for 12 h. Two nerve segments were cut: one 5 mm proximal and one 5 mm distal to the chitin conduit (Figure 2, blue segments). After this step, each sample was stained in 1% osmium tetroxide for 12 h and then dehydrated through a graded series of ethanols, and the specimens were then immersed in xylene, embedded in paraffin, and sliced into 5 *μ*m cross-sections. Images were acquired under a microscope, from which the total number of myelinated axons and myelin thicknesses and diameters was evaluated.

**Figure 3:**
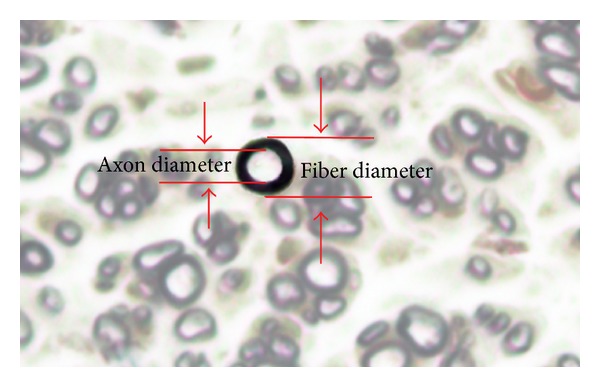
Morphometric measurement method.

Morphometric measurements were performed using ImageJ software. The shortest lengths of the outer and inner margins of the myelin sheath were measured to determine the fiber diameter and axon diameter [5] (Figure 3). After obtaining the fiber and axon diameter, myelin thickness was calculated.

### 3.5. Statistical Analysis

One-way analysis of variance was employed to compare the number of myelinated nerve fibers, the TFI, the MNCV, and the morphometric measurements (e.g., the fiber diameter, axon diameter, and myelin thickness) in all groups. *t*-test was employed to compare the amplification ratio in the two surgical groups. A probability of *P* < 0.05 was considered significant for all statistical comparisons. All values are presented as the mean ± SD.

## 4. Results

### 4.1. General Observations

**Figure 4:**
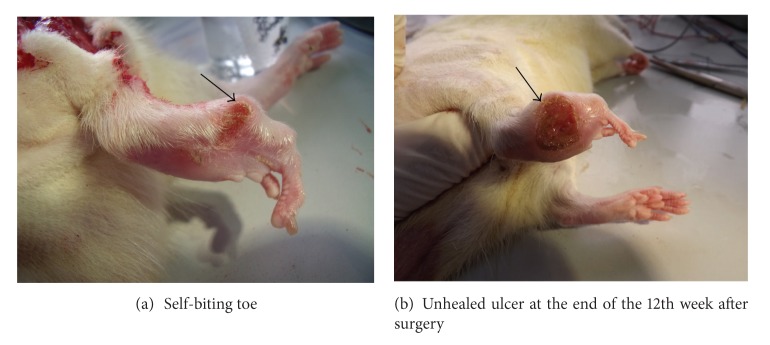
Toes on the operated side.

Animals in the model group and the treatment group were found to have postoperative lameness in the operated limbs, characterized by sagging ankles and awkward movements. The limbs of two rats appeared swollen and ulcerated 1 week after operation, and, 3 weeks later, the number of rats with ulcers increased and three rats exhibited the toe self-biting phenomenon (Figure 4(a)). The motor function of the operated limbs began to gradually recover 4 weeks after surgical operation, and most ulcers gradually disappeared and eventually healed. However, at the end of the 12th week after surgery, the ulcers of two rats in the model group had still not healed (Figure 4(b)).

### 4.2. Tibial Function Index (TFI)

**Figure 5:**
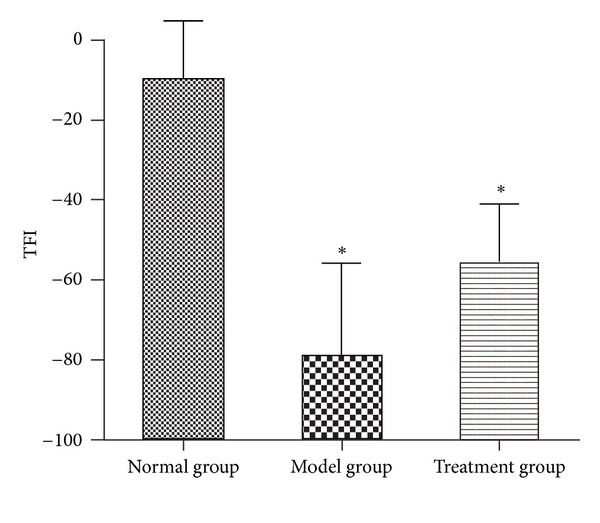
Tibial function index (TFI). *P < 0.05 versus normal group.

Walking track analysis of rats that did not have ulcers and toe self-biting showed that TFI in the normal group, model group, and treatment group was −9.29 ± 14.44, −79.06 ± 23.12, and −56.44 ± 14.55, respectively. The value in the normal group was significantly higher compared with the model group and the treatment group (*P* < 0.05). The values in the model group and the treatment group were not significantly different (*P* > 0.05) (Figure 5).

### 4.3. Motor Nerve Conduction Velocity (MNCV)

**Figure 6:**
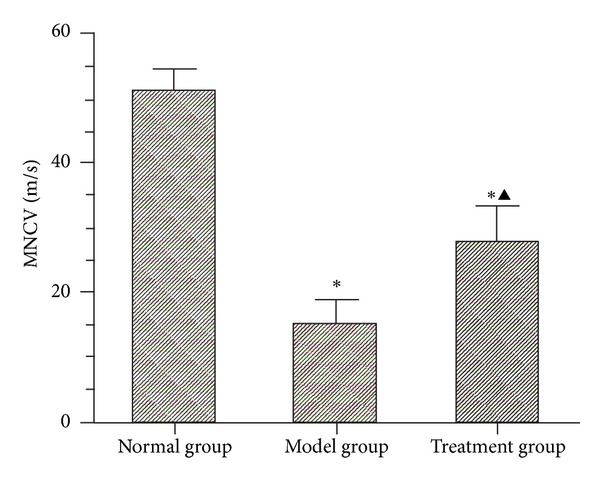
Motor nerve conduction velocity (MNCV). *P < 0.05 versus normal group; ^▲^ *P* < 0.05 versus model group.

Electrophysiological assessment was conducted prior to sacrificing the animals 12 weeks after surgery. MNCV in the normal group was 51.23 ± 3.46 m/s, the value in the model group was 15.30 ± 3.71 m/s, and the value in the treatment group was 27.88 ± 5.80 m/s. MNCVs in the normal group and the treatment group were obviously better than in the model group (*P* < 0.05). The value in the treatment group was obviously lower than in the normal group (*P* < 0.05) (Figure 6).

### 4.4. Osmium Tetroxide Staining Results

**Figure 7:**
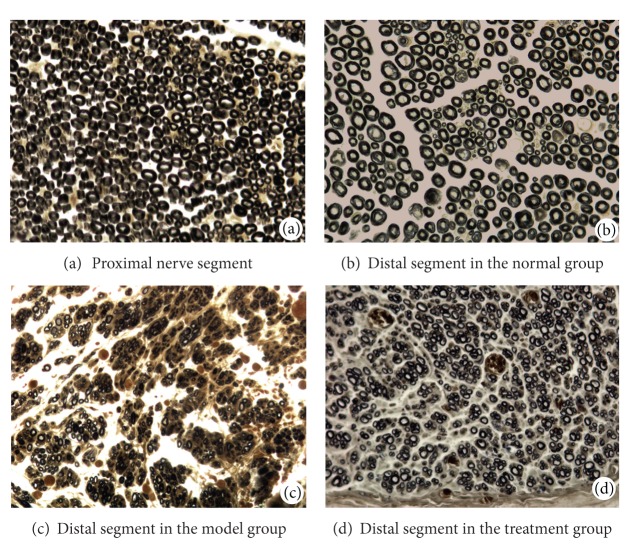
Osmium tetroxide staining in each group.

Twelve weeks after surgery, the sectioned nerves from each group were stained with osmium tetroxide. In the proximal nerve segments in the normal group (Figure 7(a)), good myelin regeneration with a uniform distribution was observed, and osmium tetroxide staining showed that the diameters and thicknesses of the myelin sheathes were uniform and regular. In the distal segments in the normal group (Figure 7(b)), the characteristics were similar to those of the proximal segments. In the distal segments in the model group (Figure 7(c)), myelin regeneration was poor, with an uneven distribution and a low density. The myelin sheathes were highly variable, and osmium tetroxide staining showed that their diameters and thicknesses were uneven and generally smaller than those in normal tissue. In addition, the myelin sheathes were irregularly shaped and some myelin degeneration and necrosis could be seen. In the distal segments in the treatment group (Figure 7(d)), myelin regeneration was better than in the model group but poorer than in the normal group. The myelin distribution was not very uniform, but their density was higher than those in the model group. The diameters and thicknesses of the myelin sheathes were generally smaller than those in normal tissue, but the diameters were larger than those in the model group. A low level of myelin degeneration and necrosis was observed.

Distal nerve segments, 5 mm distal to the chitin conduits, were measured and analyzed in animals from each group. As shown in Table 1, fiber diameter in the normal group and treatment group was significantly larger compared with the model group (*P* < 0.05), and the value in the treatment group was significantly lower compared with the normal group (*P* < 0.05). The differences in axon diameter between any two groups were all significant (*P* < 0.05), the value in the normal group and treatment group was significantly larger compared with the model group, and the value in the treatment group was significantly lower compared with the normal group. Myelin thickness in the model group and the treatment group was significantly lower compared with the normal group (*P* < 0.05), and the values in the model group and treatment group were not significantly different (*P* > 0.05). 

### 4.5. Amplification Ratio for Nerve Regeneration

As shown in Table 2, the amplification ratio for nerve regeneration in the model group was 1.47 ± 0.19, which was obviously less than that in the treatment group (1.96 ± 0.09; *P* < 0.05).

## 5. Discussion

Previous studies have reported an amplification phenomenon during nerve regeneration. In the initial stages of nerve regeneration after injury, one of the proximal nerve fibers grew several lateral buds, and these lateral buds grew into the distal myelin sheath tube [6]. According to this phenomenon, a relatively fine nerve can be used as a donor to repair the distal injured nerve in clinical situations. The donor nerve can grow more collaterals that extend to the distal stump, thus ensuring the structural and functional recovery of damaged nerves at a lower cost. This study further confirmed that there is an amplification effect during nerve regeneration.

Distal nerves undergo Wallerian degeneration after peripheral nerve injury [7]. In this process, distal axons and myelin degenerate and collapse and Schwann cells proliferate and form Bungner tubes in the original endoneurium to provide growth channels for the regenerating axons. Meanwhile, Schwann cells secrete a variety of neurotrophic factors and adhesion molecules that induce and promote newborn lateral buds to grow into the distal endoneurium tube [8]. Each axon in a proximal stump can grow several buds at the Ranvier knot close to the stump [1, 9]. Generally during the regeneration period, when the sprouts are blocked by scar tissue, connective tissue, and other kinds of obstructions, or when they grow in the wrong direction, they cannot grow into the distal endoneurial tubes and gradually degenerate. Therefore, when axons establish terminal structures, the number of collaterals is reduced to the usual single trunk [10]. However, when the number of distal endoneurial tubes is more than that of donor axons (i.e., to say, the number of distal fibers is more than that of donor fibers), they can provide sufficient space for regenerating axons to grow into, and thus the nerve fiber amplification effect arises. In this study, the small peroneal nerve was treated as a donor nerve to connect to the distal tibial nerve via a chitin absorbable tube, and the results verified the nerve regeneration amplification effect.

This amplification phenomenon may provide a new method for the clinical repair of nerve damage, thus enabling the repair of nerve defects at a lower cost and the induction of nerve regeneration by a small gap bridge that is compliant with nerve growth law. The bridging method is superior to traditional epineurium suture and perineurium suture, but it does not yet effectively promote functional recovery. We therefore hypothesized that an adjuvant therapy given after surgical operation may induce lateral bud growth by proximal axons, promote the amplification effect, and eventually achieve functional recovery. In this study, we administered MFRH to rats as an adjuvant therapy.

Several studies have demonstrated that MFRH can promote regeneration after peripheral nerve injury. Radix Hedysari is a major component of this prescription, and it can effectively improve the motor nerve function index, nerve conduction velocity, and the number of regenerated myelinated nerve fibers [3]. *Lumbricus* which is another component of the prescription can also improve functional recovery following injury by increasing the total number of regenerated myelinated nerve fibers in adult rats [4], indicating that it has potential clinical therapeutic value for peripheral nerve injury. The regeneration of damaged nerves is a multifactorial process involving a variety of mechanisms, cells, and factors [11–14]. Any treatment method using a single factor for adjuvant therapy is obviously one-sided and may not be effective in targeting all pathological mechanisms involved in peripheral nerve injury. Recent studies have shown that locally applied neurotrophins can enhance the survival of damaged neurons and induce regrowth of lesioned axons in the central and peripheral nervous systems in rats [15], but the beneficial effect is limited. Local application of neurotrophins after the peripheral nerve sleeve bridging operations has been reported by several authors. Madduri and Gander [16] advocated the inclusion of neurotrophic factors (NTFs) in the nerve conduit wall. Sun et al. [17] implanted microspheres containing NGF into the neural tube before bridging the transected peripheral nerve, but found that there was no significant difference compared with the control group three months later with respect to nerve conduction velocity, muscle tension, and muscle wet weight. Therefore, local applications that do not treat the body as a whole do not achieve the desired therapeutic effectiveness. Traditional Chinese Medicine may provide a more favorable growth microenvironment for nerve regeneration by inducing various biologically active factors required by the process. In this study, we chose MFRH as an adjunctive treatment for the peripheral nerve amplification model. The results showed that this prescription played a positive role in promoting the amplification of peripheral nerves. General observations revealed that ulcers in two animals in the model group did not heal within 12 weeks postoperatively, while all of the ulcers in the animals in the treatment group had healed. We speculated that the prescription may exhibit neurotrophic effects and stimulate immune function to promote wound healing. However, an increased sample size is required to further confirm these functions of MFRH. Statistical analysis revealed that although the TFIs in the treatment group and the model group were not significantly different, the value in the treatment group was higher compared with the model group (*P* = 0.133). From the results, we speculate that treatment with MFRH may promote the functional recovery of damaged peripheral nerves. Because the number of animals used for walking track analysis was limited, excluding animals (with ulcers and toe self-biting) which could not leave complete footprints for analysis, an increased sample size is required for subsequent experiments to further confirm the functional recovery effects of MFRH. Statistical analysis also revealed that the nerve conduction velocity in the treatment group was 27.88 ± 5.80 m/s, which was significantly higher than that in the model group (15.30 ± 3.71 m/s; *P* < 0.05). The fiber diameter and axon diameter in the treatment group were larger compared with the model group (*P* < 0.05), while myelin thicknesses in the two surgical groups were not obviously different (*P* > 0.05). The nerve regeneration amplification ratio in the treatment group was 1.96 ± 0.09, which was obviously higher than that in the model group (1.47 ± 0.19), and the difference was statistically significant (*P* < 0.05).

These experimental results confirmed the positive role of MFRH in the amplification of peripheral nerves, thus providing a new method for the clinical repair of peripheral nerve injury. The mechanism by which MFRH promotes the amplification effect is multifactorial. At present, it is known that Schwann cells play an important role in the nerve repair process as they constitute the main component of myelin sheaths and secrete many important factors essential for peripheral nerve regeneration. MFRH can promote the proliferation of Schwann cells through the “receptor—cyclic adenosine monophosphate (cAMP)—protein kinase A (PKA) signaling pathway,” and it can also promote the differentiation of Schwann cells via receptor tyrosine kinase (RTK) signaling pathways [18]. By regulating Schwann cell proliferation and differentiation, this prescription promotes the repair of injured peripheral nerves. As reported in another study, the prescription promotes nerve regeneration by promoting the secretion of neurotrophic factors such as bFGF, NGF, and Trk [19], and these neurotrophic factors play complex and positive roles in peripheral nerve regeneration and repair. Macrophages have a neuroprotective capacity with a sterile inflammatory response and participate in tissue repair after acute peripheral nerve injury [20]. Hedysari (containing Hedysari Polysaccharides (HPS)) can significantly promote the activity of macrophages [18] that can effectively clear debris, caused by degenerated nerve and myelin, to facilitate nerve regeneration. Immune cells and immune factors play a positive role in regeneration after peripheral nerve injury. HPS could potentially improve the ratio of *α*-naphthol acetate esterase-positive lymphocytes and improve the transformation rate of *in vivo* lymphocytes induced by phytohemagglutinin, demonstrating mediation effects to T-lymphocyte cell-mediated immune function [21] and showing another mechanism to promote nerve regeneration and repair. Following peripheral nerve injury, the activation of multiple intracellular signaling cascades causes the expression of regeneration-associated transcription factors, which interact with each other to determine the fate of injured neurons. Transcription factors form a vital link in the chain of regeneration, converting injury-induced stress signals into downstream protein expression via gene regulation [22]. MFRH may promote the expression of transcription factors and is awaiting exploration at the genetic level. HPSs, the main active ingredients in Radix Hedysari, have a protective effect on the inner cell membrane barrier [23], can improve the cytotoxicity of lymphokine-activated killer cells (LAK cells) and peripheral blood mononuclear cells (PBMCs) [24], and also reduce free radicals by enhancing the activity of SOD and GSH-px [25]. All of these effects suggest that the mechanism by which the prescription promotes nerve regeneration and amplification effect is multifaceted, because Traditional Chinese Medicines are multicomponent complexes that may provide appropriate conditions similar to the nerve regeneration microenvironment.

A previous study that used different donor nerves of different diameters to dominate the distal tibial nerve [26] showed that each surgery group exhibited the amplification effect, with all parameters being better than those in the control group. Tibial nerve function index and tibial nerve conduction velocity decreased gradually with a decrease in donor nerve fibers, because the gradual reduction in the number of donor nerve fibers caused the absolute number of regenerating nerve fibers to reduce even though each surgery group exhibited an obvious amplification effect. This study concluded that when the number of donor nerve fibers was half the number of the original injured nerve fibers, the best structural and functional recovery was observed [26]. Another conclusion from this study was that the amplifying ratio maximum was 3.3 [26]. Our observations showing that MFRH can effectively promote the amplification effect should be explored further in subsequent experiments. For example, when the prescription is used as adjuvant therapy, are structure and function best recovered if fewer donor nerve fibers (less than half of the distal fiber number) are used to repair and dominate the distal tibial nerve, and will the amplification ratio maximum be greater than 3.3?

In this study, we investigated the effect of MFRH at one time point only (12 weeks after surgery). Further studies are therefore required to explore its long-term effect.

## Figures and Tables

**Table 1:** Morphometric measurements in different groups 12 weeks after surgery.

Group	Fiber diameter (*μ*m)	Axon diameter (*μ*m)	Myelin thickness (*μ*m)
Normal group	8.20 ± 1.82	3.60 ± 0.88	2.30 ± 0.70
Model group	3.75 ± 0.85*	1.75 ± 0.64*	1.00 ± 0.28*
Treatment group	5.35 ± 0.88^∗▲^	2.95 ± 0.60^∗▲^	1.20 ± 0.25*

*P < 0.05 versus normal group; ^▲^ *P* < 0.05 versus model group.

**Table 2:** The number of myelinated fibers and amplification ratio in each group.

Group	Number of proximal fibers	Number of distal fibers	Amplification ratio
Normal group	4984.27 ± 294.07	5060.25 ± 466.30	—
Model group	2016.94 ± 181.38	2954.83 ± 334.08	1.47 ± 0.19
Treatment group	1953.75 ± 132.18	3819.01 ± 265.76	1.96 ± 0.09*

*P < 0.05 versus model group.
